# Face Mask in COVID-19 and Its Association With Dry Eye Disease: A Cross-Sectional Study

**DOI:** 10.7759/cureus.32937

**Published:** 2022-12-25

**Authors:** Rohini Motwani, Siddharam S Janti, Vidya Ganji, Kalpana R Mali, Kishore Yadav, Nabnita Patnaik, Arvind Kumar Morya

**Affiliations:** 1 Anatomy, AII India Institute of Medical Sciences, Bibinagar, Hyderabad, IND; 2 Ophthalmology, All India Institute of Medical Sciences, Bibinagar, Hyderabad, IND; 3 Physiology, AII India Institute of Medical Sciences, Bibinagar, Hyderabad, IND; 4 Pharmacology, All India Institute of Medical Sciences, Bibinagar, Hyderabad, IND; 5 Community and Family Medicine, AII India Institute of Medical Sciences, Bibinagar, Hyderabad, IND; 6 Obstetrics and Gynaecology, All India Institute of Medical Sciences, Bibinagar, Hyderabad, IND

**Keywords:** sars cov, n-95, health care workers, face mask, dry-eye, covid-19

## Abstract

Aim: To determine whether wearing a face mask for more than three to six hours/day leads to the new onset of symptoms or worsening of pre-existing dry eye disease (DED) in healthcare workers (HCWs) of our institute.

Methodology: An observational cross-sectional study, where 114 HCWs using face masks regularly participated voluntarily in the study. A survey with a modified Ocular Surface Disease Index (OSDI) questionnaire was completed by participants. They were divided into groups based on their sex, age, how long they had been wearing face masks, and whether they had a history of DED.

Results: We found that for HCWs who had previously experienced DED and who were under the age of 40, wearing a face mask for more than three to six hours/day could contribute to or worsen symptoms of DED. Also, we observed that the N-95 mask has a higher chance of causing DED than surgical masks.

Conclusion: Medical professionals need to be aware of any potential dry eye symptoms related to the prolonged use of a face mask. Additional consideration should be given to patients who already have DED. The possible concerns that incorrectly fitted facemasks may cause to the health of their ocular surface should be discussed with patients by ophthalmologists. Future research involving larger populations will shed light on the prevalence and scope of the mask-associated dry eye problem.

## Introduction

December 2019 has been remarkable in everyone’s life due to the emergence of a new disease named Coronavirus Disease 2019 (COVID-19) caused by SARS-CoV-2, which was first detected in Wuhan (Hubei Province, China) [1]. COVID-19 is a respiratory infection that is transmitted by direct contact and respiratory droplets through coughing, sneezing, breathing, and talking. Personal protective equipment (PPE) [2], particularly face masks (FMs), play a vital role in preventing COVID-19 infection in addition to routine social distancing and hand and surface hygiene practises due to the high transmission rate of COVID‐19 [3] and predisposition for airborne infection [4]. All these measures have now become part and parcel of our lives, and most of us have accepted and called it a ‘new normal’ change. Government regulatory bodies recommended the use of FM, especially in public places, workplaces, crowded areas, or restricted environments where physical separation is insufficient to protect oneself and others from droplet infection [5]. Although the benefits of wearing FM are still up for debate, the development of regulatory guidelines has led to a significant increase in the use of face masks, especially in crowded areas or congested circumstances where maintaining physical space is challenging [5-6]. However, long-term use of FMs has been linked to a number of side effects, including increased airway resistance, pressure and pain in the nose and ears, changes in the temporomandibular joint, and itch and discomfort in the eyes [7-8].

The use of masks for an extended period of time has been linked to problems like headaches, breathing difficulties, skin irritation, sweating, and fogged glasses [9-10]. Mask-associated dry eye (MADE) was first observed anecdotally in June 2020, when White, an American ophthalmologist, described the condition on his blog and coined the acronym 'MADE' [11]. Emerging research suggests that during the COVID-19 pandemic, there was an increase in the prevalence of ocular symptoms with continued use of FMs, and while using FMs, the upward flow of air during expiration or the limited movement of the lower eyelid promotes faster tear evaporation and hence, the beginning or aggravation of dry eye disease (DED)-related symptoms [12-13]. An additional risk of post-operative mask-associated dry eye due to poorly fitted FM (MADE) was reported after cataract surgery under topical anaesthesia [14].

The Tear Film and Ocular Surface Dry Eye Workshop II (TFOS DEWS II) report defines DED as a multifactorial ocular surface disease where tear film instability and hyperosmolarity, ocular surface inflammation and damage, and neurosensory abnormalities play etiological roles and are accompanied by ocular symptoms and a loss of tear film homeostasis [15]. Ocular pain, dryness, itching, and a feeling of a foreign body are among the symptoms of DED [16]. Accurate DED assessment benefits from the evaluation of subjective DED symptoms using patient-reported outcome questionnaires (with the Ocular Surface Disease Index (OSDI) being the most frequently utilised) in addition to clinical findings [6,17].

In the past few months, ophthalmologists at our institute have noticed an increase in the new onset of complaints related to dry eye or the worsening of symptoms in those who already had DED, which may be related to wearing FMs for a prolonged period. Healthcare workers (HCWs) must care for patients while also protecting themselves from infection. Hence, it is mandatory for them to wear face masks, which are typically worn for extended periods of time. At present, the data on DED due to FM use are very limited, and we did not find any studies specifically on HCWs. Therefore, we planned to investigate whether wearing FM for an extended period could cause irritation of the eye or worsen DED symptoms.

## Materials and methods

This study was reviewed and approved by the Institutional Research Board (IRB) and Institutional Ethics committee (IEC) of the institute independently. The study adhered to the principles of the Helsinki Declaration. In this cross-sectional study, out of 120 HCWs employed in the institute, 114 HCWs who participated voluntarily were included in the study after receiving informed written consent.

Objective

The main objective of the study was to determine whether wearing FM for more than three to six hours/day leads to the new onset of symptoms of DED or worsening of pre-existing DED in HCWs at our institute.

Inclusion criteria

All the male and female healthcare workers working in the healthcare system at the All India Institute of Medical Sciences (AIIMS), Bibinagar, wearing a surgical mask, N-95 masks, or other types of masks, and those participants with and without DED, were included in the study.

Exclusion criteria

Participants who had recently experienced active ocular surface conditions other than DED, such as episcleritis or conjunctivitis, allergies, a history of using antihistamine or hypotensive eye drops, prior intraocular or refractive surgery in either eye, neurologic or autoimmune conditions (such as Parkinson's disease or Bell's palsy), etc., were excluded from the study.

Participants were given a pre-tested questionnaire containing a modified OSDI [18] questionnaire prepared in three different languages, namely English, Hindi, and Telugu, which they filled out according to their understanding. The first part of the questionnaire contained demographic details, the type of mask preferred, and the duration of daily mask wear. The second part included a 12-item questionnaire for assessing the various symptoms and measuring the subjective severity of DED. The score of the OSDI is on a scale of 0-100. The higher the OSDI score, the more severe the dryness [19]. Participants in the study completed the survey on their own or with the assistance of investigators as needed (especially elderly participants).

Participants were divided into two groups based on gender, based on age (younger than 40 years and 40-60 years), duration of wearing FM (<3 hours, three to six hours, and >6 hours per day), and prior DED history. Following survey completion, the OSDI index was calculated using the formula OSDI = (sum of scores) × 25/(number of questions answered), and the severity of DED was graded as mild (1-22), moderate (23-32), and severe (>33) [20].

An ophthalmological examination was done for the HCWs, which included best-corrected visual acuity, measurement of intraocular pressure, and slit lamp assessment of the ocular anterior segment. The DED was confirmed by clinical tests such as the Schirmer test, tear film break-up time (T-BUT), corneal fluorescein staining (FS), and lissamine green staining (LS). TBUT was recorded as the average of three consecutive measurements, and less than 10 seconds in either eye was considered to be dry eyes. The corneal FS has been evaluated with 1% sodium fluorescein and scored 0 to 3 according to the Oxford grading scale [21], whereas the LS was graded according to the classification proposed by Sullivan et al. [22].

Statistical analysis

Statistical analysis was done using SPSS software version 22 (IBM Corp., Armonk, NY), and the normality of the data was checked by the Shapiro-Wilk test. All the descriptive data were expressed as percentages, medians, and interquartile ranges. Mann-Whitney U tests and Kruskal-Wallis tests were used as tests of significance. Dunn's test was applied for post-hoc analysis. Statistical significance was set at P < 0.05.

## Results

This study was done on 114 HCWs, including 61 doctors, 16 nurses, and 37 multi-tasking skilled workers (class IV employees) working at AIIMS Bibinagar, Hyderabad. Of the 114 participants, 51 (44.7%) were male and 63 (55.3%) were female HCWs and 77% of the participants belonged to age <40 years and 23% belonged to 40-60 years. Of note, most of the participants (77%) used masks for more than six hours/day, 18% of the subjects used masks for three to six hours/day and 4% of them used masks for <3 hours/day. Most of the HCWs were using surgical masks (47.4%) and N-95 masks (46.5%) (Table 1). Out of 114 HCWs, 24% had prior DED symptoms before using the mask, and the most common symptoms reported were eye grittiness, dryness, and scratchiness, followed by eye fatigue or burning sensation. Of note, 76% of the participants did not have any symptoms of DED before using the mask. The most common symptom reported by these HCWs after using FM was dryness, grittiness, and scratchiness, followed by eye fatigue, soreness, and irritation of the eyes. The severity of dryness was mild in 35% of the subjects who were using N-95 masks without respirators and moderate in 8% of the subjects.

**Table 1:** Distribution of subjects according to age, sex, occupation, and type of mask used (n=114).

	Frequency	Percent
Age		
<40 years	88	77.2
40–60 years	26	22.8
Sex		
Male	51	44.7
Female	63	55.3
Occupation		
Doctors	61	53.5
Nurses	16	14.0
Class IV	37	32.5
Type of mask		
Cloth	1	0.9
N95 with Respirator	4	3.5
N95 without respirator	53	46.5
Surgical mask	54	47.4
Others	2	1.8

**Figure 1:**
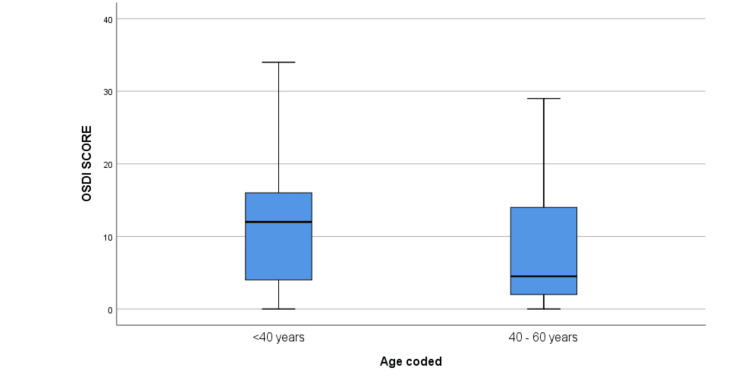
Comparison of Ocular Surface Disease Index score in different age groups. Statistical significant difference was observed between the mean ranks of group aged below 40 years and group aged 40-60 years (P = 0.03).

**Figure 2:**
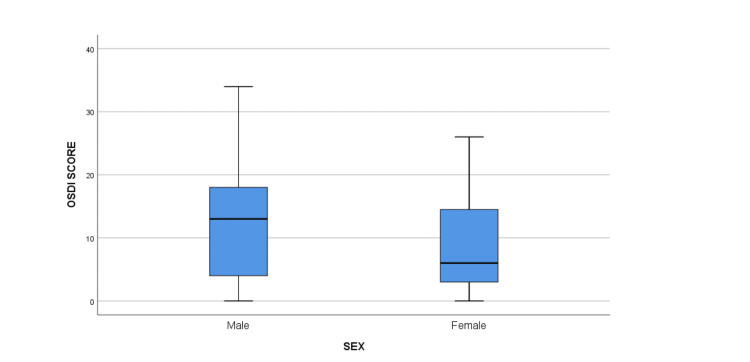
Comparison of OSDI score in different genders. The mean rank of OSDI score of males had higher than female group but the difference was not statistically significant (p = 0.140). OSDI: Ocular Surface Disease Index.

**Figure 3:**
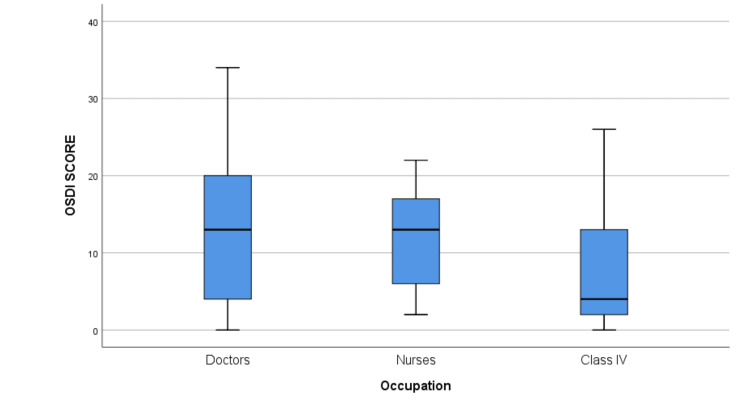
OSDI score differences according to usage of face masks in specific categories of healthcare workers as per their occupation. Significantly higher OSDI score was noted in doctors and nurses. OSDI: Ocular Surface Disease Index.

The median OSDI score was significantly higher among participants in the <40-year age group when compared to the 40-60-year group (12 [IQR: 4, 16]) (p = 0.03) (Table 2) (Figure 1). The median OSDI score in men was higher compared to women, but the difference was not statistically significant (p = 0.140) (13 [IQR: 4, 18]) (Figure 2). The median OSDI scores were significantly higher in doctors (20 [IQR: 4, 20]) followed by nurses (17 [6, 17.5]) when compared to class IV HCWs (p-value = 0.004) (Figure 3). The OSDI scores were higher in subjects with a duration of mask usage >6 hours (14 [IQR: 8, 17]) followed by three to six hours a day (11 [IQR: 6, 13]), and the OSDI score was significantly higher in HCWs with prior DED before using a mask (p = 0.005) (Figure 5). The OSDI score was highest (67.24) in 53 participants who were using N95 masks without respirators compared to the OSDI score (48.29) in 54 participants who were using surgical masks. Pairwise comparison by Dunn’s post-hoc test of OSDI scores across occupations showed a statistically significant difference between the mean ranks of doctors and class IV employees (P = 0.006) and Nurses and Class IV employees (P = 0.043). Pairwise comparison by Dunn’s post-hoc test of OSDI scores across the duration of mask usage showed a statistically significant difference between the groups that used face masks for <3 hours and three to six hours (P = 0.017).

**Table 2:** Median OSDI score of subjects based on age, sex, occupation, and duration of mask usage (n=114). OSDI: Ocular Surface Disease Index.

Variable	Median OSDI score	Inter quartile range	P-value
Age			
<40 years	12	4, 16	0.03
40–60 years	4.5	1.75, 14.5	
Sex			
Male	13	4, 18	0.14
Female	6	3, 15	
Occupation			
Doctors	20	4, 20	0.004
Nurses	17	6, 17.5	
Class IV	4	2, 13	
Duration of mask usage			
<3 hours	3	0, 10	0.03
3–6 hours	11	6, 13	
>6 hours	14	8, 17	

**Figure 4:**
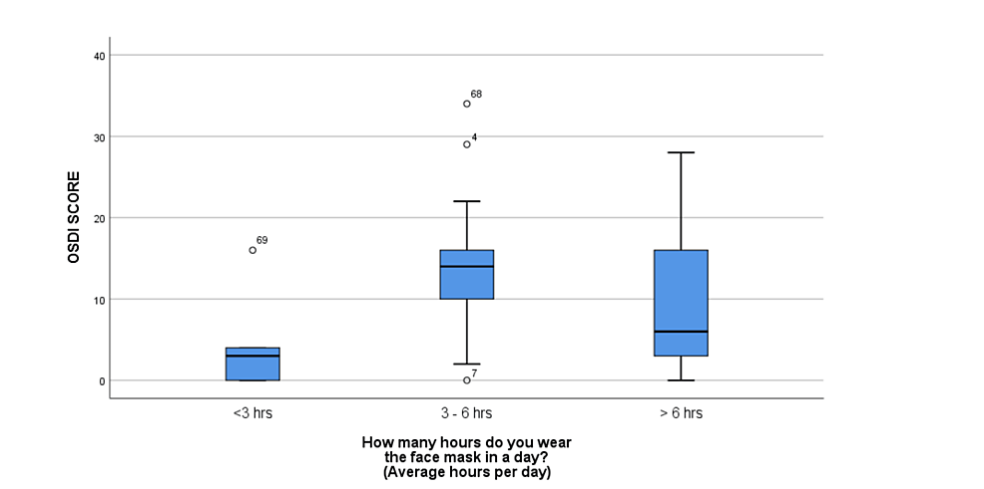
OSDI score differences according to duration of face mask wearing. Significantly higher OSDI score was noted in the group wearing masks for >6 hours compared to 3-6 hours and 0-3 hour/day group. OSDI: Ocular Surface Disease Index.

**Figure 5:**
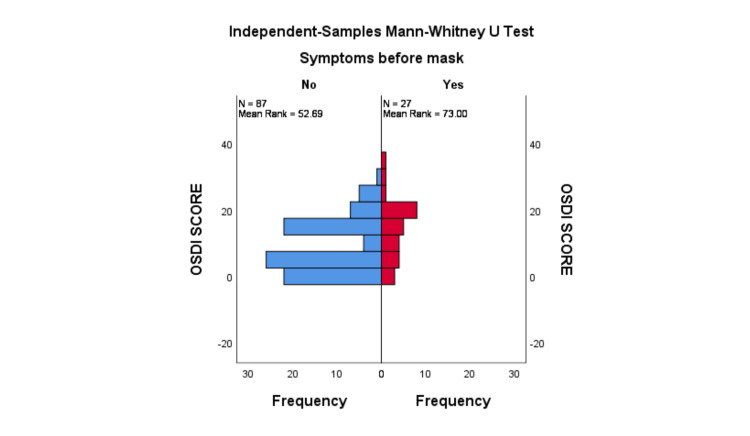
OSDI score according to previous dry eye disease history. Prior dry eye disease group had significantly higher OSDI score. OSDI: Ocular Surface Disease Index. N: number of healthcare workers 87 with new onset symptoms of DED, 27 prior DED symptoms present.

## Discussion

According to our observations, participants under the age of 40 had a statistically higher OSDI score than those between the ages of 40 and 60, and men had a higher OSDI score than women. Whereas in another study, the OSDI score was higher for female participants in comparison to male participants [23]. In a similar study on HCWs, no significant differences were observed in DED between genders [24]. The OSDI values showed a significant difference between age groups, with the age group <40 years having a high median value. The TFOS DEWS II report confirmed that DED prevalence increases significantly and has a linear relationship with age, with signs increasing more than symptoms per decade [25]. There was a significant difference in the median rank of the OSDI score of doctors and nurses in the present study when compared to other groups, and wearing FM for more than six hours a day resulted in a significant difference in the OSDI score. In a study by Scalinci et al. [26] on those utilising FM for at least six hours per day and at least five days per week, a significant increase in the OSDI score was seen. Contrarily, Krolo et al. found that patients who used a face mask for three to six hours each day had significantly higher OSDI scores than those who wore the mask less frequently. Additionally, compared to other groups, daily mask use for a longer period of time did not produce a significant OSDI score. The reason suggested was that most of the participants who were wearing FM >6 hours/day were younger and healthier and were wearing masks during their working hours [23].

**Figure 6:**
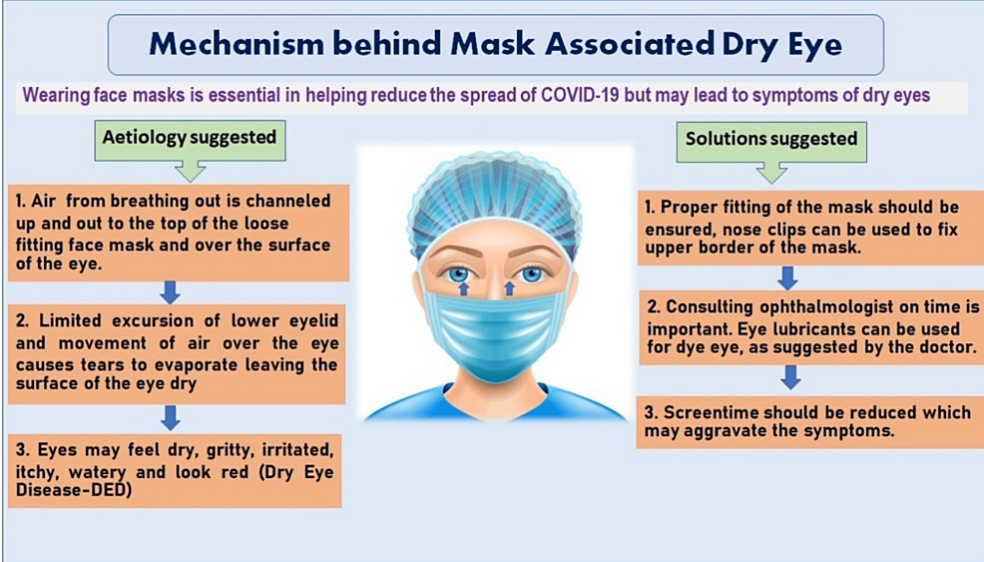
Showing mechanism of mask-associated dry eye disease and solutions or suggestions to prevent dry eye disease arising due to use of mask (created by authors Dr Rohini Motwani and Dr Vidya Ganji).

Now the question is: if using an FM for an extended period of time results in the worsening of the symptoms of DED, the pathophysiological cause has yet to be determined. In general, unless the subject is exercising, respirators and surgical masks should not modify the blood gas composition [27]. Blood gas composition is also unlikely to have an impact on how severe DED symptoms are. The most common explanation for the worsening of DED symptoms could be masks that do not fit snugly around the nose. Respiratory gases may be directed upwards and towards the eyes in the absence of a proper seal (Figure 6). The microenvironment inside face masks, and consequently the types of gases that are diverted upwards, can be different from that of ambient air because these masks do not readily dissipate air. This is particularly true if FM with a respirator and without an exhaust valve is used [26]. The temperature and humidity of the gases inside an FM have been found to be higher according to earlier studies; this can also be a factor in the worsening of DED [28].

Mastropasqua et al. conducted clinical examinations, in vivo confocal microscopy (IVCM), and impression cytology to evaluate the dry eye-related quality of life score (DEQS) and described the ocular surface alterations brought on by an FM (IC) in their study. They suggested that the chronic use of FMs may increase the chance of developing dry eyes in healthy individuals, while in DED patients, it greatly increases the risk of ocular surface deterioration. When used regularly throughout the day, FMs harm the ocular surface in the presence of DED, causing a significant worsening of several clinical and molecular parameters. The authors also emphasised that FM use increases ocular surface inflammation and has a negative impact on the quality of life of these patients [29]. In the present study, 24% of HCWs had prior DED symptoms before using the mask, and the most common symptoms reported were eye grittiness, dryness, and scratchiness, followed by eye fatigue or burning sensation. The OSDI score was significantly higher in HCWs with prior DED before using the mask. In another study, authors reported worsening symptoms in patients with prior DED during the period for wearing a mask regardless of the duration for which he/she wears a mask [23]. Although diminished corneal sensitivity and neurosensory abnormalities are now identified as common symptoms of DED [15], there have also been reports of disruptions in the neuronal networks of the cornea that lead to hypersensitivity [30].

The OSDI score was highest (67.24) in 53 participants wearing N95 masks without respirators, compared to 48.29 in 54 participants wearing surgical masks in our study. In a cross-sectional study on HCWs for assessment of DED in N95 versus surgical FM wearers during COVID‑19, authors observed a significant increase in the OSDI score in the N95 mask group versus the surgical mask group [24]. But if we look into the theory proposed, it is surprising as N-95 FM is more air-sealed, so the chances of air skipping must be less as compared to surgical or other masks. Further studies are definitely recommended to confirm these subjective observations.

To the best of our knowledge, very few studies are available to date assessing the correlation of wearing an FM (surgical mask or N-95) with dry eye symptoms. Although using an FM for an extended period of time may exacerbate DED symptoms, the authors of this study would never advise against using an FM. Instead, authors urge regular use of FM for the protection of themselves as well as the community. The results of this study are crucial because dry eye symptoms can cause irritation of the eyes, affect the ocular surface, and, in prolonged scenarios, affect the quality of life of patients.

Limitations of the study

This study included only HCWs, as they wear the FM regularly to protect themselves from COVID-19 infection and for longer duty hours. The subjective nature of the OSDI score in this study may be considered one of the limitations. More research involving larger populations is encouraged.

Recommendations

Since HCWs who frequently interact with patients or who work in crowded environments cannot avoid FM, it is crucial that they are aware of the signs and symptoms of dry eye and seek medical attention as soon as possible to stop further deterioration. Additionally, we advise users who are experiencing worsening DED symptoms to either increase the frequency of installations or the density of eye lubricants. Taping the area around the nose may also be helpful. In fact, tape assists in creating an airtight seal that hinders the entry of respiratory gases to the ocular surface.

## Conclusions

With this study, we can conclude that wearing FM for more than three to six hours per day with prior DED, especially in males of age less than 40 years, could contribute to the worsening of the symptoms of DED. Also, we observed that the N-95 mask has a higher chance of causing DED than surgical masks. Medical professionals need to be aware of any potential dry eye symptoms related to the prolonged use of FM. Additional consideration should be given to patients who already have DED. Ophthalmologists should inform their patients about the potential risks that improperly fitted facemasks may pose to the health of their ocular surface. It takes little time and can make a big difference to ask patients about their experiences wearing masks and give a few helpful tips. Future research involving larger populations will shed light on the prevalence and scope of the dry eye problem associated with the mask.
